# Short-term efficacy and safety of lasmiditan, a novel 5-HT_1F_ receptor agonist, for the acute treatment of migraine: a systematic review and meta-analysis

**DOI:** 10.1186/s10194-020-01138-x

**Published:** 2020-06-05

**Authors:** Min Hou, Haiyan Xing, Chen Li, Xianfeng Wang, Dongmei Deng, Juan Li, Pan Zhang, Jianhong Chen

**Affiliations:** Department of Pharmacy, Daping Hospital, Army Medical University, Chongqing, 400042 P. R. China

**Keywords:** Migraine, Lasmiditan, 5-HT_1F_ receptor agonist, Meta-analysis

## Abstract

**Background:**

Migraine has been recognized as one of common diseases in the world whose current treatment options are not ideal. Lasmiditan, an oral 5-hydroxytryptamine (HT)_1F_ receptor agonist, appears more promising for the acute treatment of migraine because of considerably better effect profiles with no severe adverse events (AEs). This review aimed to systematically evaluate the efficacy and safety of lasmiditan from the results of randomized controlled trials (RCTs).

**Methods:**

PubMed, Cochrane Library, Embase were searched on lasmiditan for the acute treatment of migraine from inception of the databases to Feb 1, 2020. Pain free and pain relief, global impression (very much/much better), and no/mild disability at 2 h in efficacy; total treatment-emergent adverse events (TEAEs), dizziness, nausea, fatigue, paraesthesia and somnolence in safety were extracted from the included studies. A systematic review and meta-analysis was performed using Review Manager Software version 5.3 (RevMan 5.3).

**Results:**

Four RCTs with a total of 4960 subjects met our inclusion criteria. The overall effect estimate showed that lasmiditan was significantly superior to placebo in terms of pain free (RR 1.71, 95% CI 1.55–1.87), pain relief (RR 1.40, 95% CI 1.33–1.47), global impression (very much/much better) (RR 1.55, 95% CI 1.44–1.67), and no/mild disability (RR 1.15, 95% CI 1.10–1.20) at 2 h. For the safety, significant number of patients experienced TEAEs with lasmiditan than with placebo (RR 2.77, 95% CI 2.53–3.03), most TEAEs were central nervous system (CNS)-related and included dizziness (RR 5.81, 95% CI 4.72–7.14), nausea (RR 2.58, 95% CI 1.87–3.57), fatigue (RR 5.38, 95% CI 3.78–7.66), paraesthesia (RR 4.48, 95% CI 3.33–6.02), and somnolence (RR 2.82, 95% CI 2.18–3.66).

**Conclusions:**

This meta-analysis suggests that lasmiditan is effective for the acute treatment of migraine with a higher incidence of CNS-related adverse reactions compared with placebo. Long-term, open-label, multi-dose trials are required to verify the current findings.

## Background

Migraine is a common neurological disease that was ranked by the Lancet Global Burden of Disease Study as the second highest cause of disability in 328 diseases from 195 countries between 1990 and 2016, and is becoming a larger component of the global burden of disease [1]. Statistically, 45.1 million of total years lived with disability are suffered from migraine [1], which has a significant impact on quality of life and increased use of health resources [2, 3]. It is characterized by moderate-severe, unilateral, throbbing headache attacks lasting from 4 to 72 h, accompanied by additional symptoms such as nausea, vomiting, phonophobia, and/or photophobia [4]. However, the exact etiology and pathogenesis of migraine currently is unclear. Thus, to find a safety, effective and highly specific medication remains a challenge and warrants further research.

In general, the choice of acute treatment is based mainly on two classes of medicines: nonspecific (analgesics and nonsteroidal antiinflammatory drugs, NSAIDs) and specific drugs (triptans and ergot derivatives) [5]. The triptans, regarded as the gold standard in the migraine therapy, are a class of selective and effective 5-hydroxytryptamine (HT)_1B/1D_ receptor agonists that have replaced ergot derivatives. However, 30% ~ 40% of treated patients do not respond to triptans that are also endowed with the risk of serious cardiovascular adverse events caused by vasoconstriction yielded by 5-HT_1B_ receptor activation [6, 7]. Therefore, a new acute therapy for migraine is urgently needed, especially for those patients unable to achieve optimal outcomes with current therapies.

Lasmiditan, also known as COL-144 and LY573144, is a novel 5-HT receptor agonist with high-affinity and selectivity for the 5-HT_1F_ receptor, which acts on the trigeminal system without causing vasoconstriction because of its low affinity for 5-HT_1B_ receptors [8]. Representing a new class of migraine medications, lasmiditan is believed to act both centrally and peripherally, and developed as an acute therapy for migraine to address significant unmet needs in patients with cardiovascular risk factors, those with stable cardiovascular disease, or patients who respond poorly to their current treatment.

The U.S. Food and Drug Administration approved lasmiditan for the acute treatment for migraine with or without aura in adults on 11 October 2019 [9]. Data from phase II and III studies showed significant efficacy and high incidence of treatment-emergent adverse reactions (TEAEs) of this molecule versus placebo in acute treatment for migraine. However, up to now, there was no systematic review that examined the efficacy and tolerability of lasmiditan. Therefore, in this paper, we performed this systematic review and meta-analysis to evaluate the safety and efficacy of lasmiditan in the treatment of acute migraine attacks.

## Methods

### Literature search and inclusion criteria

Two reviewers (MH and HYX) independently searched PubMed, Cochrane Library, Embase for articles by entering “migraine” or “headache” and “lasmiditan” or “COL-144” or “LY573144” or “5-HT_1F_ receptor agonists” as search terms. Then all articles and their reference lists were examined to expand potentially relevant articles. The bibliographic databases were searched from their respective inception to Feb 1, 2020. The articles were included in the meta-analysis if they met the following criteria: (1) included patients were adults (18–65 years of age) with migraine with or without aura which had been diagnosed according to the International Headache Society criteria (IHS) [10, 11]; (2) randomized controlled trials (RCTs) evaluating the efficacy and safety of lasmiditan for the acute treatment of migraine; (3) lasmiditan and placebo in any formulation or in any dose as treatment group and control group respectively; (4) relevant indexes of the efficacy and safety of lasmiditan were provided or could be calculated from original data in the articles. Studies were excluded when one of the following issues occurs: (1) subjects were animals; (2) interventions were drug combinations; and (3) except for RCTs, other types of trials such as cross-over designs, healthy controlled trials and self-contrast trials. Disagreement between two reviewers was settled by consensus or consultation with a third author (JHC or LC).

### Quality assessment of the included studies

The methodological quality of included studies was assessed by two independent raters using Review Manager Software version 5.3 (RevMan 5.3) provided by the Cochrane Collaboration with a seven-item scale (random sequence generation, allocation concealment, blinding of participants and personnel, blinding of outcome assessment, incomplete outcome data, selective reporting and other bias) [12]. Each of the items involved assigning a judgment of high, low, or unclear risk of material bias with lower bias indicating better quality. Detailed criteria for making judgments about the risk of bias from each of the items in the tool are available in the Cochrane Handbook [13]. Any discrepancies between two reviewers were discussed and settled by consensus or consultation with a third reviewer (XFW or LC).

### Statistical analysis

All extracted data syntheses were performed by RevMan 5.3 (Cochrane Collaboration, Oxford, England), and overall effects and safety of lasmiditan for the treatment of acute migraine were calculated by risk ratios (RRs) with 95% confidence intervals (CIs) with a fixed- or random-effect model. The heterogeneity analyses were conducted by using Chi-square test, *I*^*2*^ values smaller than 50% indicate no significant heterogeneity, and are acceptable. The fixed-effect model of analysis is then appropriate. Otherwise, the random-effect model is considered [14, 15]. In addition, representative funnel plots were not performed to detect publication bias of the meta-analysis due to the small number of RCTs.

## Results

### Selection and inclusion of studies

**Fig. 1:**
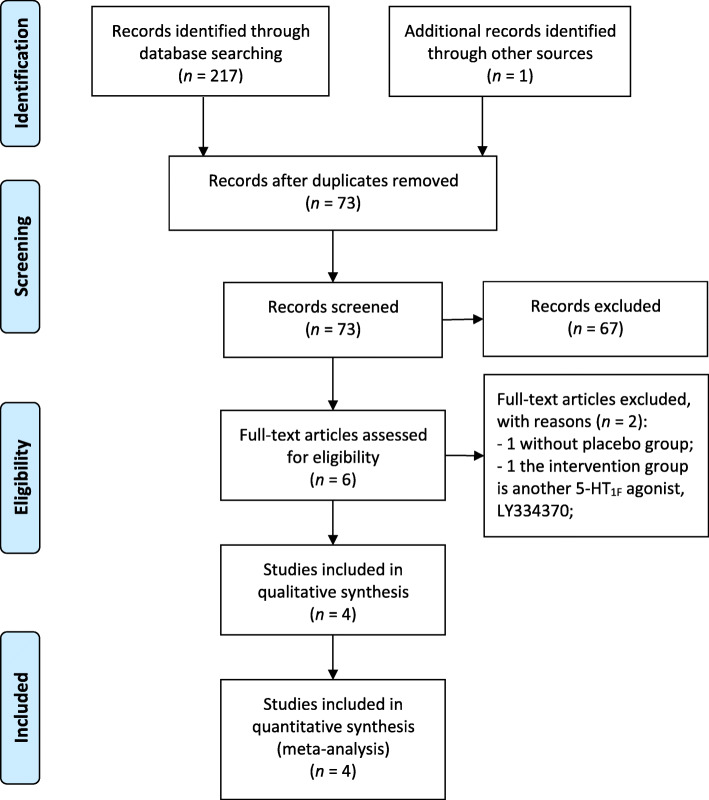
Process of identifying eligible studies for the meta-analysis

The initial search strategy retrieved 218 articles whose titles were screened for eligibility. One hundred forty-five potentially relevant studies remained after removing duplicates, then 139 reports were eliminated during abstract screening, of which full-text assessment was conducted on 6 studies. Lastly, a total of 4 RCTs involved in phase II – III (4960 participants) met the inclusion criteria and were included in this review [16–19]. A flow chart of the search strategy is shown in Fig. 1.

The baseline demographics did not differ widely among the included studies. All studies included patients with migraine classified by the IHS criteria as mentioned in the inclusion criteria. A greater percentage of subjects were female in both treatment groups: lasmiditan (84.93 ± 1.72) %, placebo (86.82 ± 2.34) %. All subjects were older than 18 years, with a mean age of 42.07 years in the lasmiditan group and 42.31 years in the placebo group. Patients had experienced a mean of 5.0 migraines per month in the lasmiditan group, and a mean of 5.1 migraines per month in the placebo group. Efficacy results were reported at primary endpoints of 2 h in placebo-controlled phase, and the safety were observed until 24 or 48 h. Details of the study characteristics were shown in Table 1.

**Table 1:** Characteristics of the included studies

Included trials	Location (s); Study design	Eligibility criteria	Gender (male/female); mean age (years)		Migraine attacks per month		Drug doses	Primary efficacy outcomes at 2 h	Most frequent TEAEs
			Control	Trial	Control	Trial			
Ferrari MD et al., 2010 [16]	Multinational; RCT	IHS 1.1 & 1.2.1	4/38; 40.3	13/75; 38.4	3.3	3.5	2.5–45 mg	Pain freedom, sustained pain free, other efficacy outcomes such as nausea, photophobia, phonophobia.	Dizziness, paresthesia, fatigue, sensation of heaviness, and feeling of relaxation
Färkkilä M et al., 2012 [17]	Multinational; RCT	IHS 1.1 & 2.1	11/75; 40.5 ± 10.3	38/267; 40.2 ± 11.0	3.1 ± 1.6	3.3 ± 1.7	50, 100, 200, 400 mg	Pain free, headache response, other efficacy outcomes such as nausea, photophobia, phonophobia	Dizziness, paresthesia, fatigue, nausea, vertigo and somnolence
Kuca B et al., 2018 [18]	USA; RCT	IHS 1.1 & 1.2.1	92/525; 42.4 ± 12.3	212/1027; 41.8 ± 11.9	5.1 ± 1.8	5.2 ± 2.1	100, 200 mg	Headache pain free, MBS free, other efficacy outcomes such as nausea, photophobia, phonophobia	Dizziness, paresthesia, fatigue, nausea, lethargy, and palpitations
Goadsby PJ et al., 2019 [19]	Multinational; RCT	IHS 1.1 & 1.2.1	100/545; 42.6 ± 12.9	309/1629; 42.7 ± 12.8	5.5 ± 2.4	5.2 ± 2.1	50, 100, 200 mg	Headac he pain free, MBS free, other efficacy outcomes such as nausea, photophobia, phonophobia	Dizziness, paresthesia, fatigue, nausea, lethargy and somnolence

*RCT* Randomized controlled trial, *IHS* The International Headache Society criteria

### Risk of bias and quality of the included studies

**Fig. 2:**
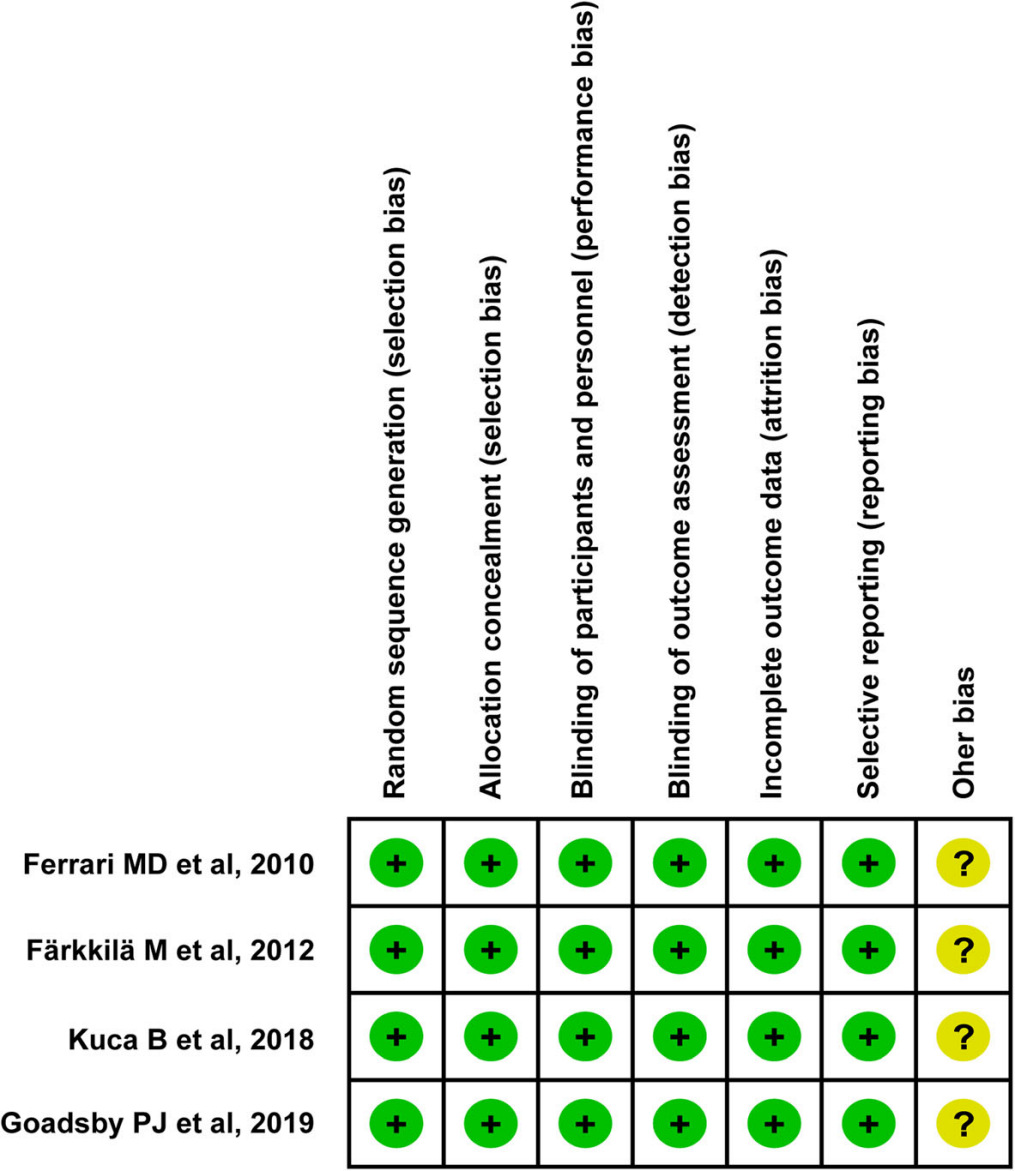
Risk of bias for included trials

Four studies evaluating the efficacy and safety of lasmiditan for migraine were included [16–19], all of which were randomized, double-blind, placebo-controlled trials. Except that the other bias was unclear, all the reviewed trials clearly described adequate random sequence generation and allocation concealment (eg. via the Interactive Response Technology system), which were evaluated as “low” risk of bias. Blinding of participants, investigators, and outcome assessors was considered adequate in all studies. Therefore, blinding of participants and personnel, and blinding of outcome assessment in all trials were classified as having a low risk of bias. Furthermore, all studies had a low risk of incomplete outcome data and selective reporting because they provided the conclusions in detail. Using the 7-item criteria in RevMan 5.3, the assessment on risk of bias between both reviewers showed an overall agreement. As presented in Fig. 2, all trials identified as low risk of bias and high-quality assessment material.

### Effectiveness of lasmiditan for the acute treatment of migraine

#### Pain free and pain relief

**Fig. 3:**
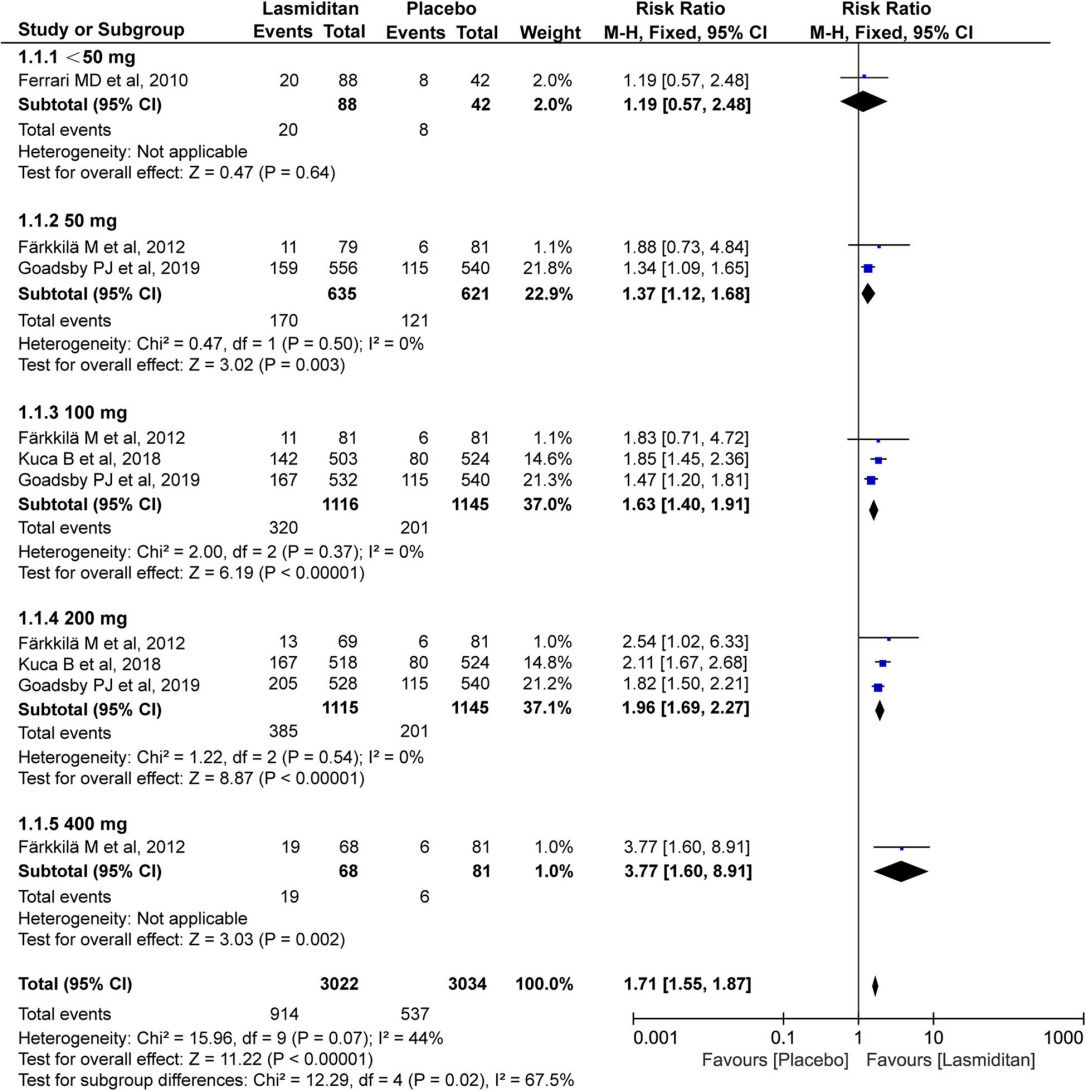
Meta-analysis of the pain free at 2 h after therapy with lasmiditan compared with placebo. The diamond indicates the estimated relative risk with 95% confidence interval for the pooled patients. M-H, Mantel-Haenszel; CI, confidence interval

**Fig. 4:**
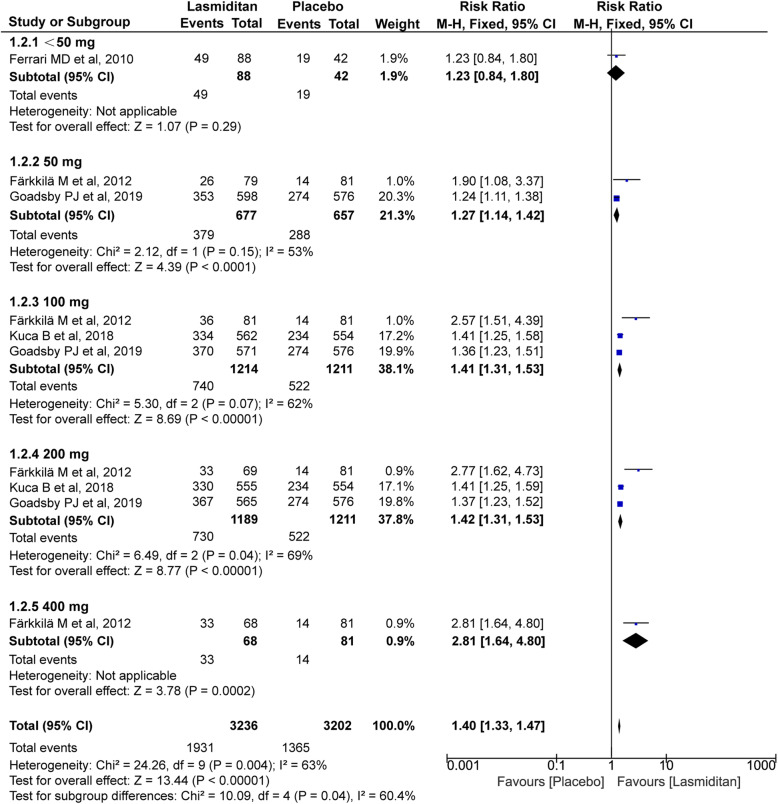
Meta-analysis of the pain relief at 2 h after therapy with lasmiditan compared with placebo. The diamond indicates the estimated relative risk with 95% confidence interval for the pooled patients. M-H, Mantel-Haenszel; CI, confidence interval

All four trials (4209 and 4489 subjects, respectively) included in this meta-analysis were evaluated for the pain free and pain relief at 2 h. As shown in Fig. 3 and Fig. 4, the significantly higher percentage of recipients treated with lasmiditan achieved pain free and pain relief after treatment compared with placebo (pain free: RR 1.71, 95% CI 1.55–1.87, *P*<0.00001; pain relief: RR 1.40, 95% CI 1.33–1.47, *P*<0.00001). Notably, there were dose-related improvements for patients who reported the pain free and pain relief across the lasmiditan treatment groups (pain free:<50 mg RR 1.19[0.57, 2.48], 50 mg RR 1.37[1.12, 1.68], 100 mg RR 1.63[1.40, 1.91], 200 mg RR 1.96[1.69, 2.27], 400 mg RR 3.77[1.60, 8.91]; pain relief:<50 mg RR 1.23[0.84, 1.80], 50 mg RR 1.27[1.14, 1.42], 100 mg RR 1.41[1.31, 1.53], 200 mg RR 1.42[1.31, 1.53], 400 mg RR 2.81[1.64, 4.80]). The *I*^*2*^ value (χ^2^ = 15.96, *P* = 0.07, *I*^*2*^ = 44%) on pain free and pain relief revealed non-significant heterogeneity among the included trials. However, there was some heterogeneity on pain relief (χ^2^ = 24.26, *P* = 0.04, *I*^*2*^ = 63%), which could result from the difference of evaluation criteria. Heterogeneity was best resolved by excluding the study by *Färkkilä M* et al (χ^2^ = 3.55, *P* = 0.62, *I*^*2*^ = 0%) [17].

#### Global impression: very much/much better

**Fig. 5:**
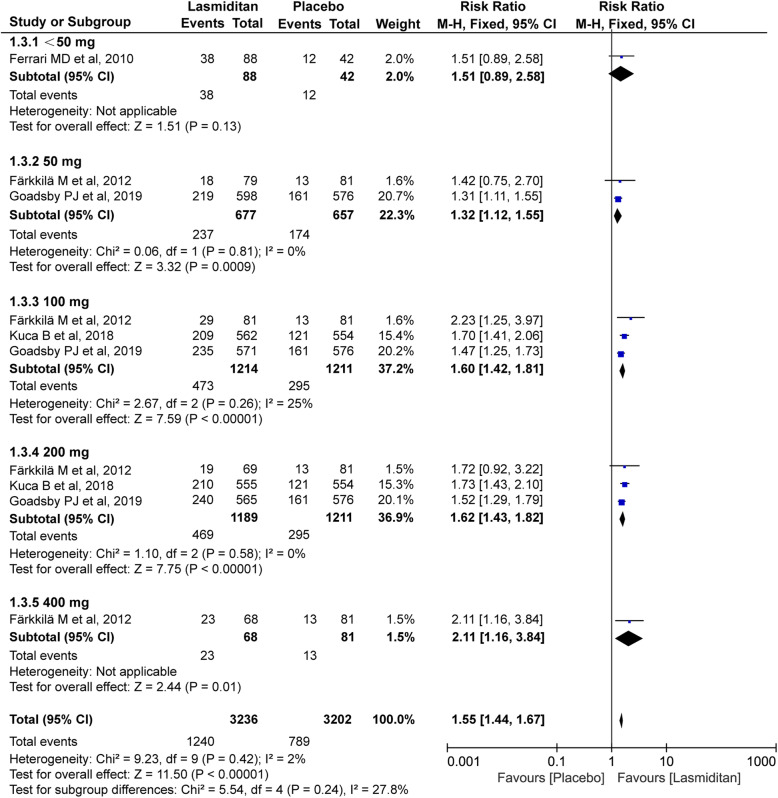
Meta-analysis of the global impression (very much/much better) at 2 h after therapy with lasmiditan compared with placebo. The diamond indicates the estimated relative risk with 95% confidence interval for the pooled patients. M-H, Mantel-Haenszel; CI, confidence interval

All four trials (4489 subjects) included in this meta-analysis were evaluated for the global impression (very much/much better) at 2 h. The overall RR after treatment favored lasmiditan over placebo (RR 1.55, 95% CI 1.44–1.67, *P*<0.00001, Fig. 5), which also had some dose-effect relation (<50 mg: RR 1.51[0.89, 2.58], 50 mg: RR 1.32[1.12, 1.55], 100 mg: RR 1.60[1.42, 1.81], 200 mg: RR 1.62[1.43, 1.82], 400 mg: RR 2.11[1.16, 3.84]). Furthermore, the *I*^*2*^ value (χ^2^ = 9.23, *P* = 0.42, *I*^*2*^ = 2%) on the global impression revealed a non-significant heterogeneity among the included trials.

#### No/mild disability

**Fig. 6:**
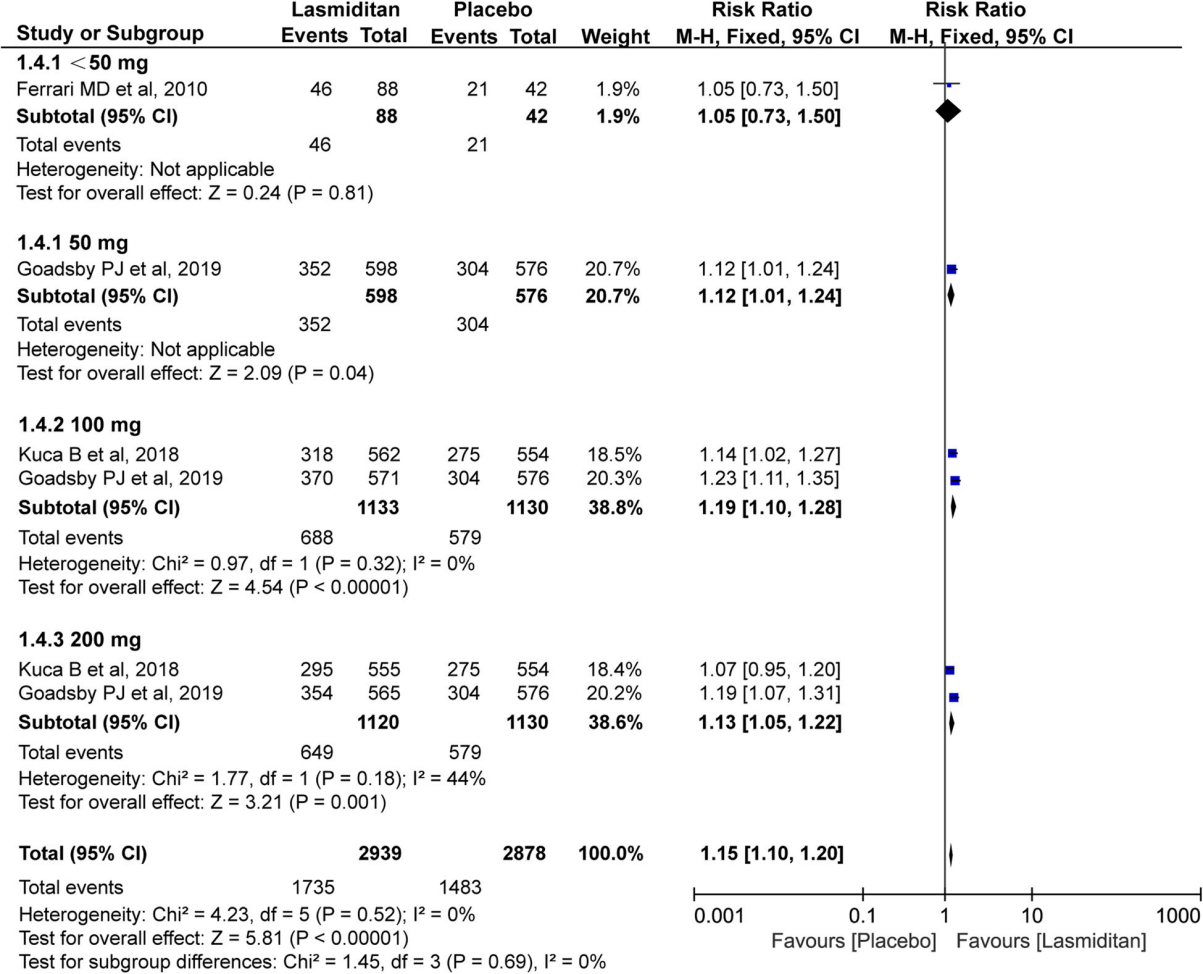
Meta-analysis of the disability (no/mild) at 2 h after therapy with lasmiditan compared with placebo. The diamond indicates the estimated relative risk with 95% confidence interval for the pooled patients. M-H, Mantel-Haenszel; CI, confidence interval

Three trials with a total of 4111 subjects included in this meta-analysis were evaluated for the no/mild disability at 2 h. As showed in Fig. 6, lasmiditan also showed benefits over placebo at 2 h in terms of the proportion of the no/mild disability patients (RR 1.15, 95% CI 1.10–1.20, *P*<0.00001). The *I*^*2*^ value (χ^2^ = 4.23, *P* = 0.52, *I*^*2*^ = 0%) on the no/mild disability revealed a non-significant heterogeneity among the included trials.

### Safety of lasmiditan for the acute treatment of migraine

#### Total TEAEs

After the first dose for 24 or 48 h, more TEAEs were reported in the lasmiditan group than in the placebo group, with a statistically significant risk ratio of 2.77 (95% CI 2.53–3.03, *P*<0.00001). Total TEAEs rate of all subgroup also had proved this dose-response relationship for the treatment of migraine (50 mg: RR 2.33[1.88, 2.89], 100 mg: RR 2.66[2.30, 3.07], 200: mg RR: 3.01[2.61, 3.48], 400 mg: RR 3.82[2.53, 5.75]) (See Additional file 1: Figure S1). Statistical heterogeneity was significant (χ^2^ = 17.33, *P* = 0.03, *I*^*2*^ = 54%), which was improved when the study by *Kuca B* et al was removed (χ^2^ = 10.17, *P* = 0.12, *I*^*2*^ = 41%) [18].

#### Main TEAEs

The most frequently reported TEAEs in migraine with lasmiditan were associated with the CNS, which included dizziness, nausea, fatigue, paraesthesia and somnolence. As shown in Table 2, there were obvious differences between lasmiditan and placebo group in these TEAEs (dizziness: RR 5.81, 95% CI 4.72–7.14, *P*<0.00001 (See Additional file 1: Figure S2); nausea: RR 2.58, 95% CI 1.87–3.57, *P*<0.00001 (See Additional file 1: Figure S3); fatigue: RR 5.38, 95% CI 3.78–7.66, *P*<0.00001 (See Additional file 1: Figure S4); paraesthesia: RR 4.48, 95% CI 3.33–6.02, *P*<0.00001 (See Additional file 1: Figure S5); somnolence: RR 2.82, 95% CI 2.18–3.66, *P*<0.00001 (See Additional file 1: Figure S6)). Furthermore, the increased risk appeared to be mostly dose-related. Majority *I*^*2*^ value revealed a non-significant heterogeneity among the included studies except dizziness (χ^2^ = 7.69, *P* = 0.10, *I*^*2*^ = 48%), which was resolved by excluding the studies by *Ferrari MD* et al [16] *and Färkkilä M* et al [17]*.*

**Table 2:** Comparison of main TEAEs between different doses of lasmiditan and placebo

Outcome or Subgroup	Studies	Participants	Risk Ratio (M-H, Fixed, 95% CI)	***P***	***I***^***2***^
**Dizziness**	4	7125	5.81 [4.72, 7.14]	<0.00001	67%
<50 mg	1	130	1.75 [0.77, 3.99]	0.18	/
50 mg	2	1467	4.55 [2.70, 7.67]	<0.00001	70%
100 mg	3	2695	5.75 [4.10, 8.06]	<0.00001	69%
200 mg	3	2677	6.59 [4.72, 9.21]	<0.00001	57%
400 mg	1	156	64.94 [4.03, 1047.06]	0.003	/
**Nausea**	3	6995	2.58 [1.87, 3.57]	<0.00001	0%
50 mg	2	1467	2.63 [1.20, 5.75]	0.45	0%
100 mg	3	2695	2.37 [1.42, 3.94]	0.0009	41%
200 mg	3	2677	2.54 [1.54, 4.21]	0.0003	0%
400 mg	1	156	13.48 [0.76, 239.65]	0.08	/
**Fatigue**	4	7125	5.38 [3.78, 7.66]	<0.00001	29%
<50 mg	1	130	1.19 [0.40, 3.58]	0.75	/
50 mg	2	1467	3.52 [1.62, 7.64]	0.001	0%
100 mg	3	2695	6.99 [3.62, 13.48]	<0.00001	0%
200 mg	3	2677	6.77 [3.51, 13.07]	<0.00001	0%
400 mg	1	156	9.83 [2.34, 41.31]	0.002	/
**Paraesthesia**	4	7125	4.48 [3.33, 6.02]	<0.00001	13%
<50 mg	1	130	20.78 [1.29, 334.92]	0.03	/
50 mg	2	1467	2.24 [0.98, 5.14]	0.06	0%
100 mg	3	2695	3.90 [2.43, 6.26]	<0.00001	21%
200 mg	3	2677	5.03 [3.17, 7.99]	<0.00001	0%
400 mg	1	156	8.60 [2.02, 36.58]	0.004	/
**Somnolence**	3	6995	2.82 [2.18, 3.66]	<0.00001	0%
50 mg	2	1467	2.86 [1.60, 5.09]	0.04	0%
100 mg	3	2695	2.59 [1.70, 3.95]	<0.00001	0%
200 mg	3	2677	2.92 [1.92, 4.42]	<0.00001	0%
400 mg	1	156	4.91 [1.08, 22.40]	0.0004	/

## Discussion

With the growing knowledge of the pathogenesis on migraine, the expression of 5-HT_1F_ receptor mRNA in neurons of the trigeminal ganglia led to the suggestion that 5-HT_1F_ receptors could be a therapeutic target for migraine [20]. As expected, it became the potential new class of anti-migraine therapy with no vascular activity and the related issues on the vascular and neuronal aspects of migraine pathogenesis. So far two selective 5-HT_1F_ agonists, LY334370 and lasmiditan, have been studied in clinical trials for the acute treatment of migraine. LY334370 was efficient with a much higher rate of asthenia, dizziness, somnolence, and parestesia than placebo for attenuating migraine attacks through selective trigeminovascular neuronal inhibition [21]. Unfortunately, the LY334370 project withdrew because of toxicity in animals [22]. Admittedly, the efficacy of LY334370 and lasmiditan also proved that vasoconstriction was not essential for anti-migraine therapy.

The U.S. FDA approval was based on positive results from two pivotal phase III trials (SAMURAI and SPARTAN), in which lasmiditan signifcantly improved the proportions of patients achieving freedom from headache pain and freedom from the most bothersome symptoms (photophobia, phonophobia or nausea) compared with placebo [9]. The current study is the first meta-analysis, to the best of our knowledge, to evaluate the efficacy and safety of lasmiditan for the treatment of acute migraine attacks. The results suggested the use of lasmiditan (daily doses from ≤50 mg to 400 mg) for patients who had at least a 1-year history of disabling migraine with or without aura was associated with significantly more pain freedom and pain relief at 2 h. Furthermore, lasmiditan also showed benefits over placebo at 2 h in terms of the proportion of patients in global impression of change ratings and disability level ratings. The findings of this systematic review confirmed that lasmiditan was superior to placebo in relieving migraine, however, as feared earlier, there was some concern about the relatively high incidence of CNS-related AEs (especially dizziness, nausea, and fatigue) as the published reviews discussing by *Peer C* et al [23] and *David K* et al [24]. The CNS-related AEs were reported in all included studies, and remarkably increased with increasing doses compared with placebo. Most adverse events affected the CNS probably due to the drugs lipophilic structure which leads to high permeability through the blood brain barrier [25], which prompted that the future development of 5-HT_1F_ agonists could give more attention to the safety profile.

For the long-term efficacy and safety of lasmiditan, a phase III GLADIATOR study involved patients who had completed SPARTAN or SAMURAI [26], and received lasmiditan 100 mg or 200 mg to be used as their frst treatment (within 4 h of pain onset) for every new migraine attack with moderate to severe pain. The interim safety and efficacy results were consistent with the previous researches, which showed a benefit of lasmiditan for reducing both the headache pain and most bothersome symptoms of migraine attacks. It is interesting to note that TEAEs over time generally showed a decrease in the incidence of these events with subsequent treated migraine attacks, and no new serious safety findings were observed, with no deaths occurring and no other trends with regard to serious AEs reported during treatment with lasmiditan for up to 1 year. Despite the most frequently reported TEAEs were associated with the CNS, there were no serious accidents or injuries resulting from a CNS-related AEs during long-term intermittent treatment. Further research should be needed to support these results, and verify the efficacy and safety of lasmiditan.

Compared to previous studies [27, 28] aimed to summarize the evidence on lasmiditan for the acute treatment of migraine, this study provided a systematic and more detailed assessment on the efficacy and safety of lasmiditan. Indeed, this first meta-analysis covered a greater number of studies and larger sample size to obtain more precise estimates on the efficacy and safety. The results showed some new valuable information about lasmiditan. First, we proved that lasmiditan (daily doses from ≤50 mg to 400 mg) was effective for the acute treatment migraine with some dose-effect relationship. Then, we analyzed the safety profile of lasmiditan by comparing TEAEs across different doses, which appeared to be mostly dose-related in the increased risk. These more detailed findings will provide some references for clinical application of lasmiditan, specially for the subpopulation of patients with relative risk factors and/or disease.

### Limitations

While this review was systematic and comprehensive, several limitations should be taken into account. First, although a total of 4960 participants were included in our meta-analysis, it was based on only four RCTs. That funnel plots were not performed to detect publication bias of the meta-analysis due to the small number of RCTs. However, all of these four trials were multicenter and high-quality RCTs. Second, the definitional standard of some efficacy and safety indicators were various and resulted in some heterogeneity in this meta-analysis, such as headache pain relief when defined as a reduction of moderate or severe pain to mild or no pain in *Färkkilä M* et al study [17], however, also included a reduction in headache severity from mild at baseline to none in *Kuca B* et al and *Goadsby PJ* et al trials [18, 19]. Third, this meta-analysis only focused on the short-term pain responses and side effects after a single dose during clinical trials and neglected the long-term efficacy and safety due to the limited data. The long-term efficacy and safety of lasmiditan remains unknown and needs to be validated following continued dosing. Furthermore, the safety evaluation period was not completely consistent in our included studies, ranging from 24 h to 48 h, which might contribute to heterogeneity. Fourth, another interesting aspect is the efficacy and tolerability of lasmiditan in patients with cardiovascular contraindications to triptans. However, the subgroup analysis was not performed due to the limited number of patients with pre-existing cardiovascular conditions in these included studies.

## Conclusions

This meta-analysis suggested that lasmiditan are effective for the acute treatment of migraine, however, with a higher incidence of CNS-related adverse reactions compared with placebo. It is critical to weigh the benefits against the risk of AEs in clinical application of lasmiditan. More long-term, open-label, multi-dose trials with larger sample sizes are needed before a definitive conclusion about the efficacy and safety of lasmiditan for migraine in the future.

## Supplementary information

**Additional file 1: **Meta-analysis of the total TEAEs and main AEs after therapy with lasmiditan compared with placebo. **Figure S1**, total TEAEs; **Figure S2**, dizziness; **Figure S3**, nausea; **Figure S4**, fatigue; **Figure S5**, paraesthesia; **Figure S6**, somnolence. The diamond indicates the estimated relative risk with 95% confidence interval for the pooled patients. M-H, Mantel-Haenszel; CI, confidence interval.

## Data Availability

All data generated or analyzed data in study are included in this article.

## Abbreviations

5-HT: 5-hydroxytryptamine
CNS: Central nervous system
IHS: The International Headache Society criteria
RCTs: Randomized clinical trials
RR: Relative risk
CI: Confidence intervals
M-H: Mantel-Haenszel
PRISMA: Preferred reporting items for systematic reviews and meta-analyses
TEAEs: Treatment-emergent adverse events
