# Supplementary material for: Short-term efficacy and safety of lasmiditan, a novel 5-HT1F receptor agonist, for the acute treatment of migraine: a systematic review and meta-analysis
Source: J Headache Pain. 2020 Jun 5;21(1):66. doi: 10.1186/s10194-020-01138-x (PMC7275414; doi:10.1186/s10194-020-01138-x)
Supplement: Supplementary file 1 — Additional file 1: Meta-analysis of the total TEAEs and main AEs after therapy with lasmiditan compared with placebo. Figure S1, total TEAEs; Figure S2, dizziness; Figure S3, nausea; Figure S4, fatigue; Figure S5, paraesthesia; Figure S6, somnolence. The diamond indicates the estimated relative risk with 95% confidence interval for the pooled patients. M-H, Mantel-Haenszel; CI, confidence interval. [file 10194_2020_1138_MOESM1_ESM.doc]

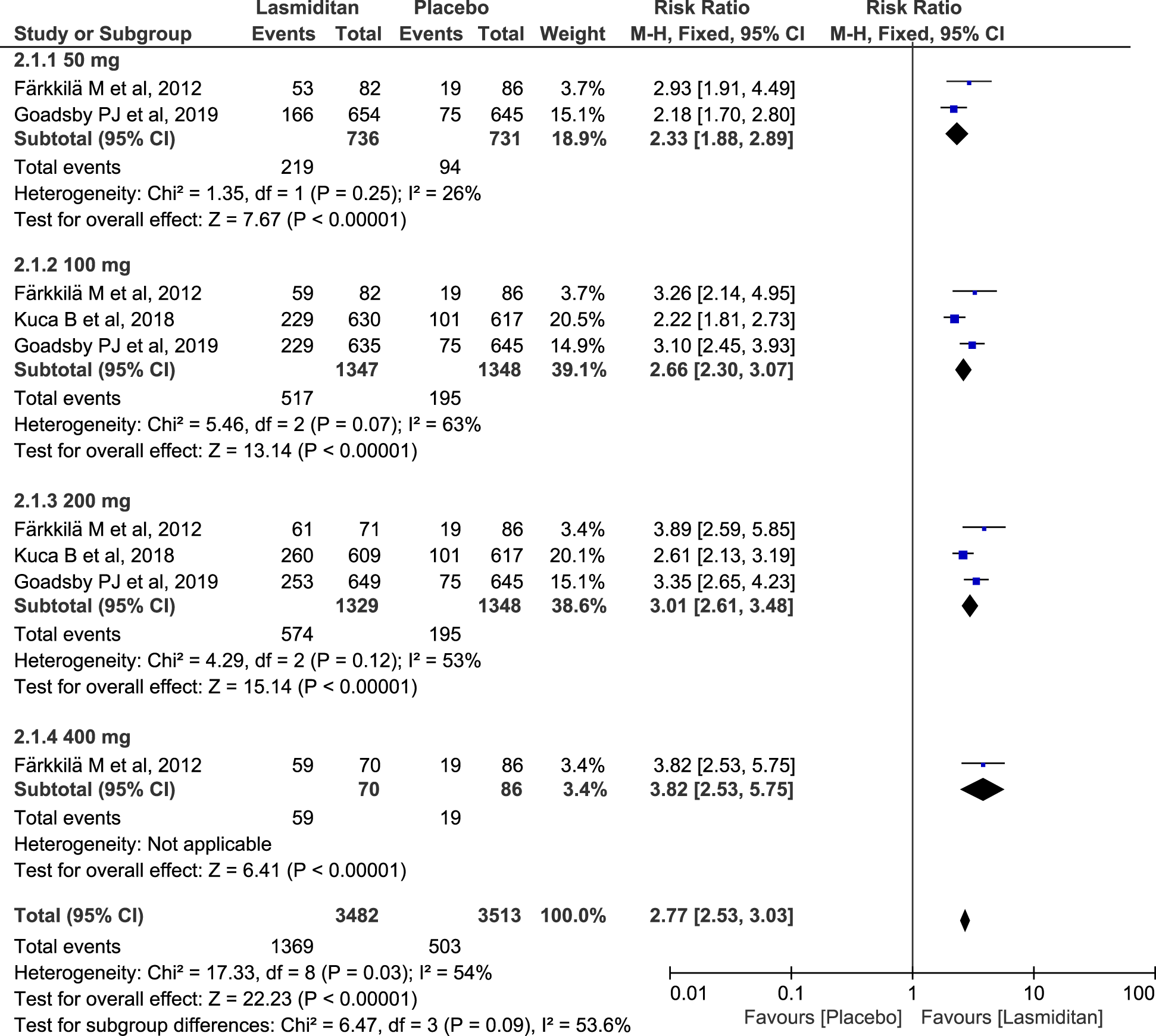


**Supplementary Figure 1** Meta-analysis of the total TEAEs after therapy with lasmiditan compared with placebo. The diamond indicates the estimated relative risk with 95% confidence interval for the pooled patients. M-H, Mantel-Haenszel; CI, confidence interval.


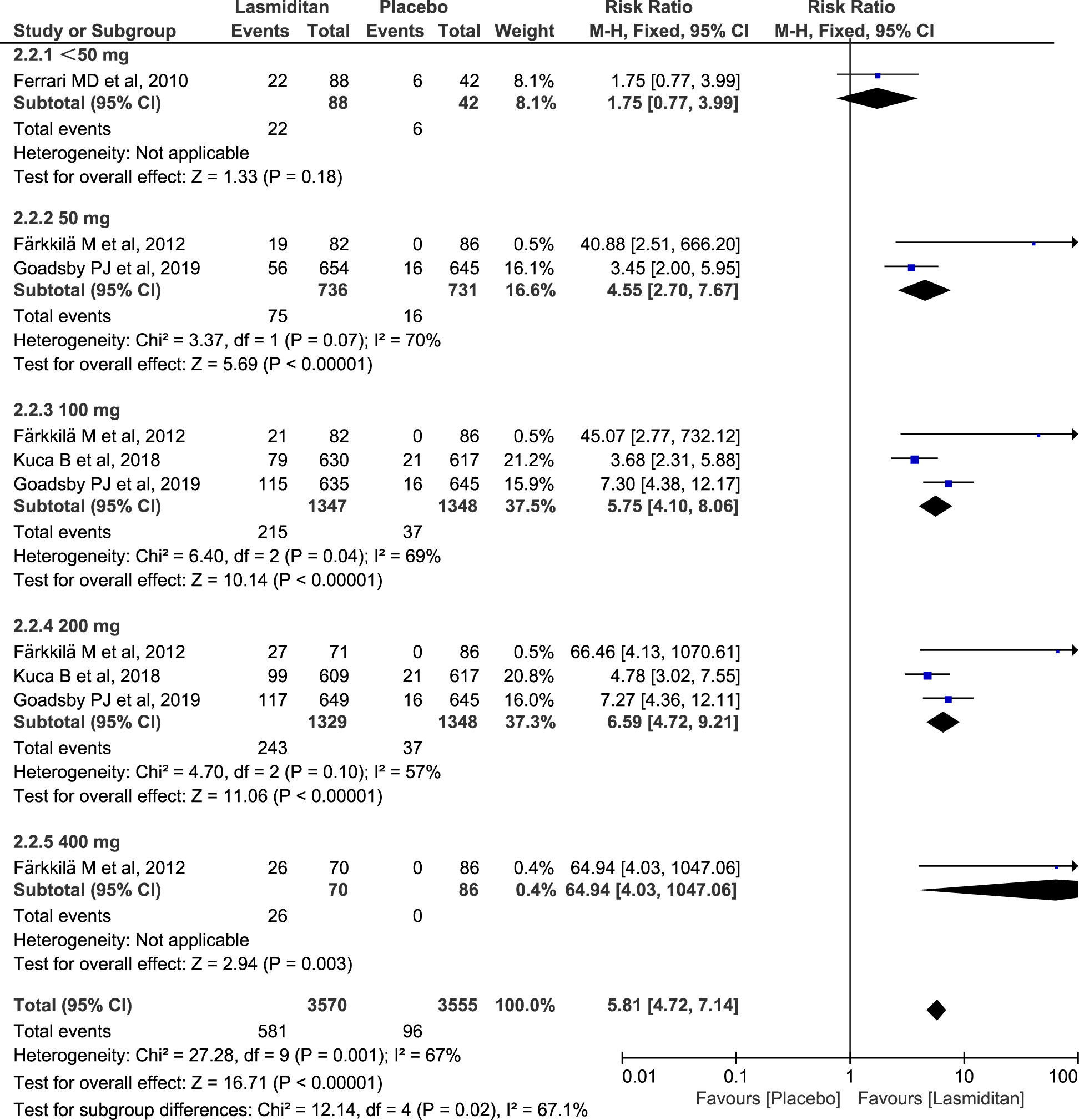


**Supplementary Figure 2** Meta-analysis of dizziness after therapy with lasmiditan compared with placebo. The diamond indicates the estimated relative risk with 95% confidence interval for the pooled patients. M-H, Mantel-Haenszel; CI, confidence interval.


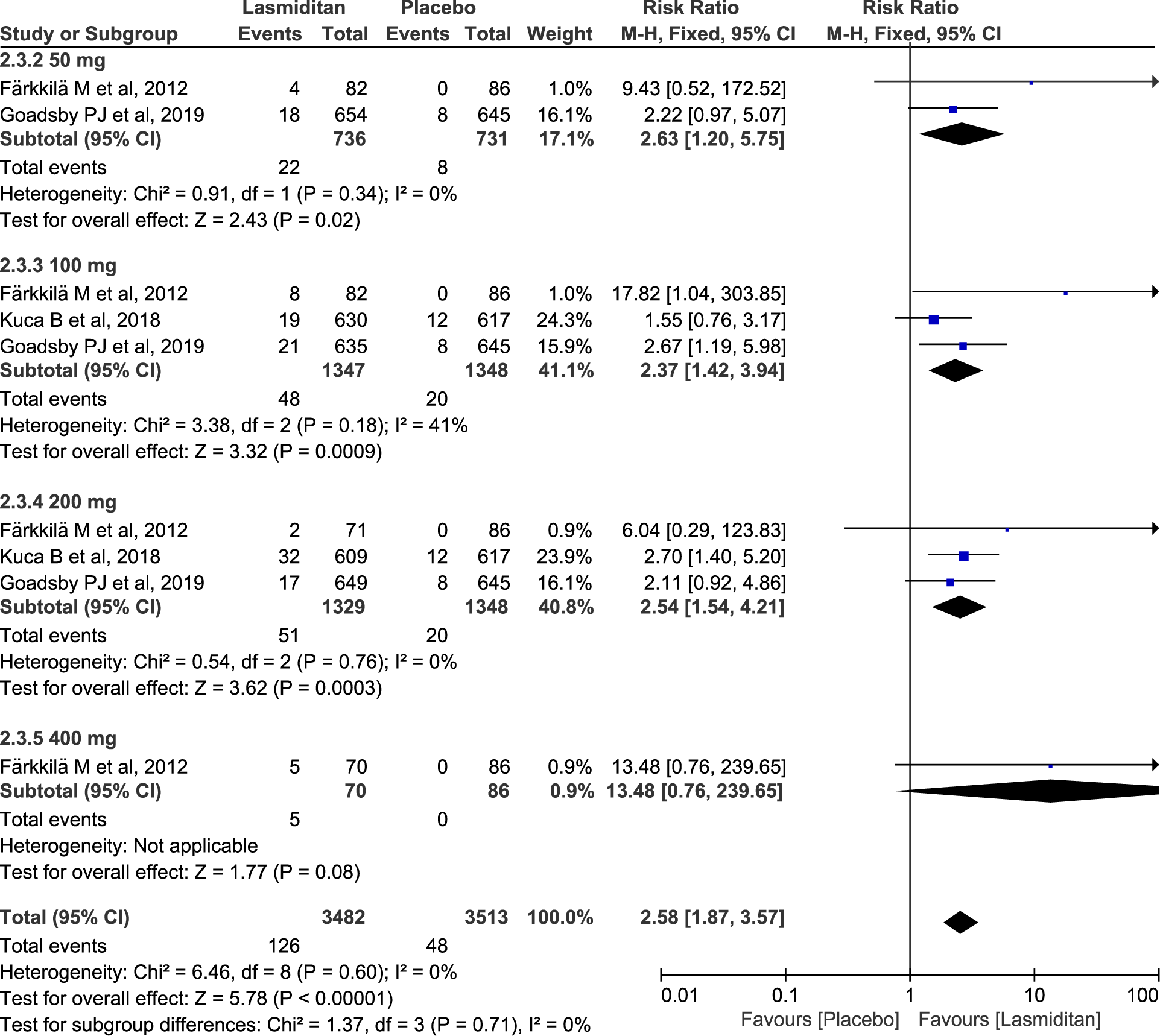


**Supplementary Figure 3** Meta-analysis of nausea after therapy with lasmiditan compared with placebo. The diamond indicates the estimated relative risk with 95% confidence interval for the pooled patients. M-H, Mantel-Haenszel; CI, confidence interval.


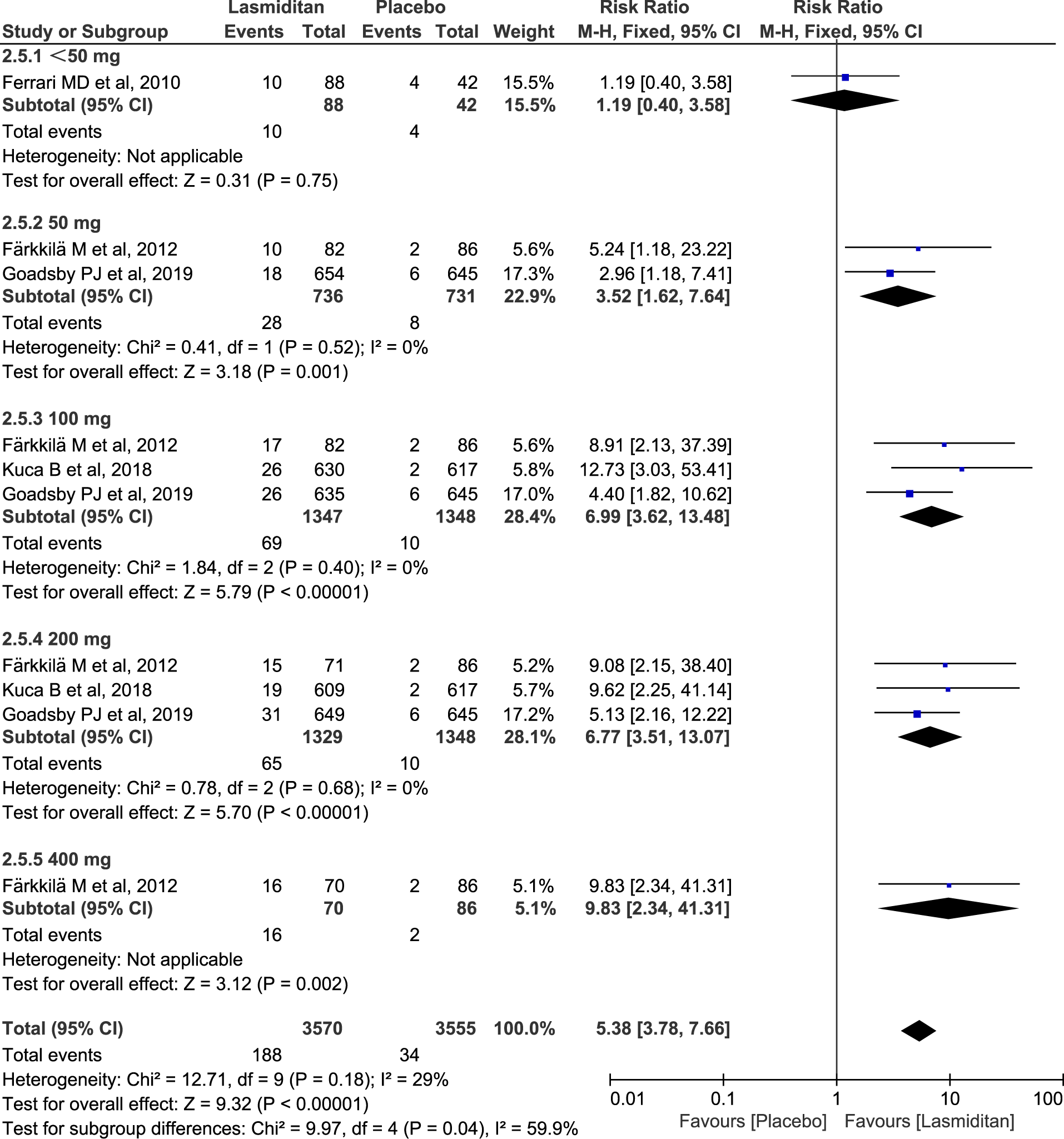


**Supplementary Figure 4** Meta-analysis of fatigue after therapy with lasmiditan compared with placebo. The diamond indicates the estimated relative risk with 95% confidence interval for the pooled patients. M-H, Mantel-Haenszel; CI, confidence interval.


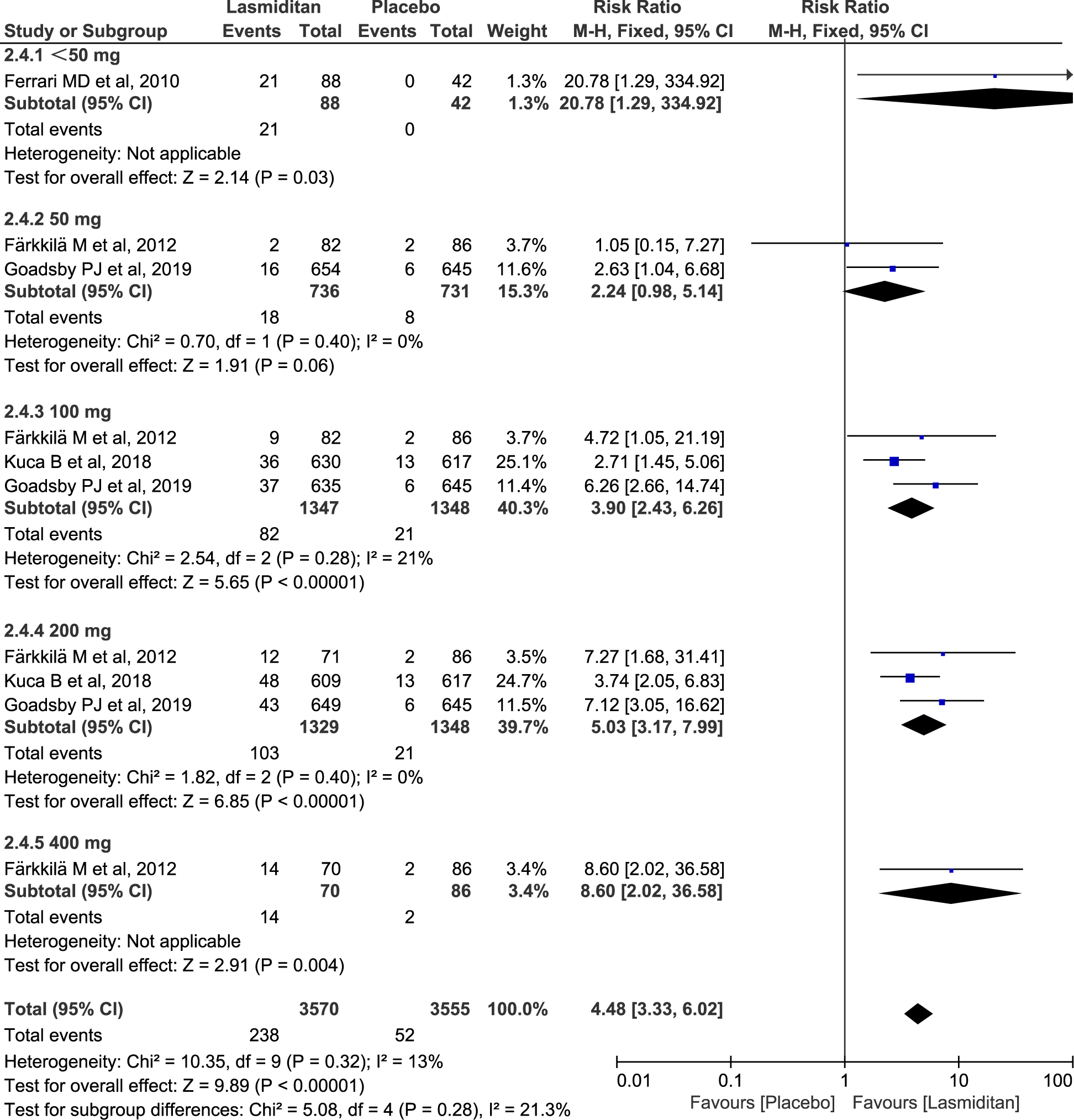


**Supplementary Figure 5** Meta-analysis of paraesthesia after therapy with lasmiditan compared with placebo. The diamond indicates the estimated relative risk with 95% confidence interval for the pooled patients. M-H, Mantel-Haenszel; CI, confidence interval.


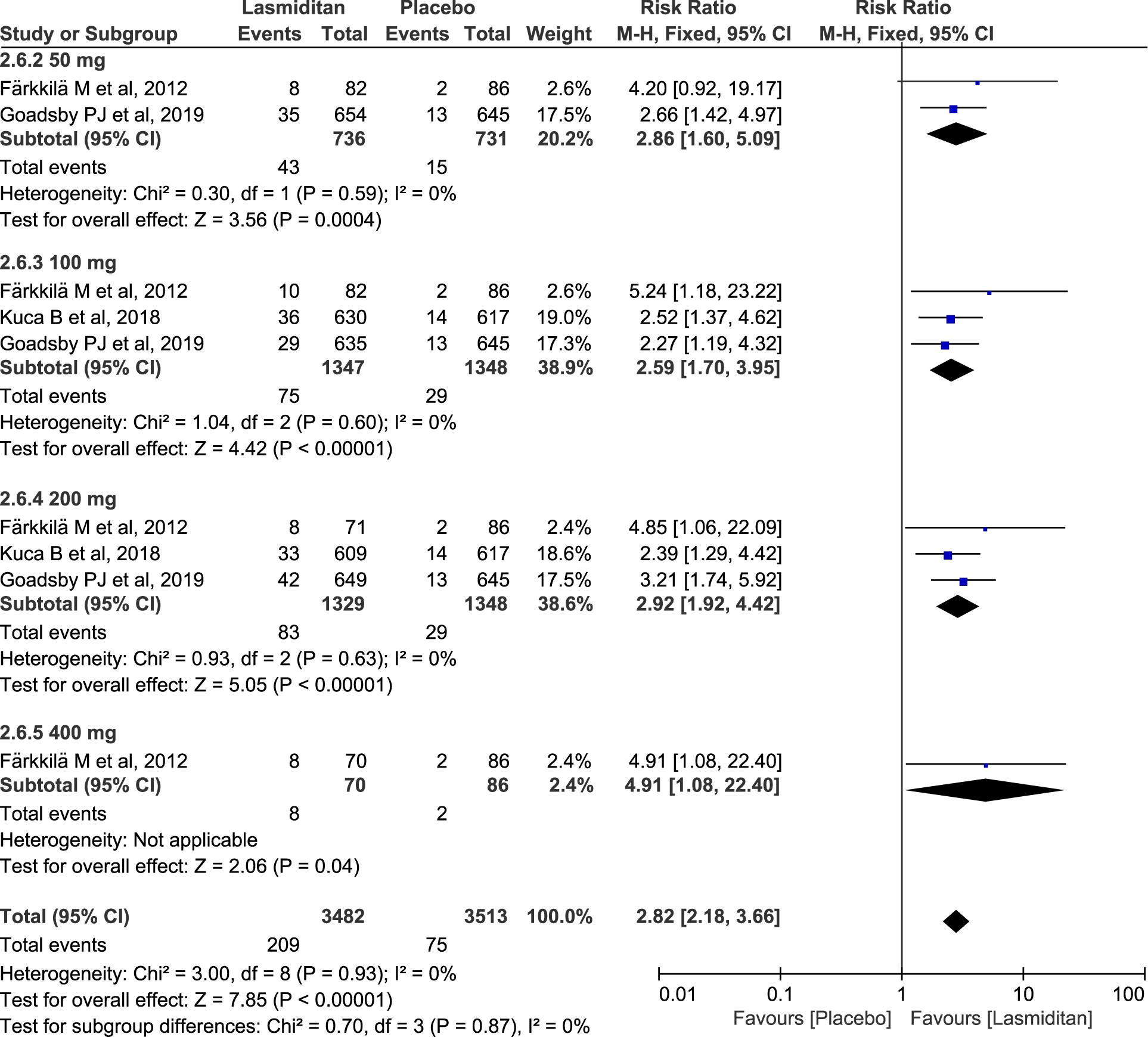


**Supplementary Figure 6** Meta-analysis of somnolence after therapy with lasmiditan compared with placebo. The diamond indicates the estimated relative risk with 95% confidence interval for the pooled patients. M-H, Mantel-Haenszel; CI, confidence interval.
